# Criminals Are Made, Not Born

**Published:** 1917-05-26

**Authors:** 


### CRIMINALITY.

### Criminals are Made, not Born.

In a previous issue we commented upon a paper by Sir Bryan Donkin in his *Journal of Mental Science* on "Mental Defect in Criminals," a paper in which the writer smashes and demolishes the hazy, groundless, self-contradictory, and absurd notions that are prevalent with respect to crime and criminals, not only in magazines, plays, novels, lectures, and newspapers, but even, and above all, in books that pretend to be "scientific" written by men who have the reputation of being "scientific." Nor are these preposterous notions limited to "scientific" writers and to the general public. They are adopted with eagerness and unction by the criminals themselves. Not long ago a prisoner, Sir Bryan tells us, pleaded in a law court that all men were irresponsible because they were unable to help doing what they did. Sir Bryan Donkin sardonically remarks that the man was pronounced after examination not to be insane, so it is to be presumed that the same opinion must be pronounced upon the "scientific" writers from whom he learnt his excuse. Another convict was so well versed in the modern literature of "criminology" that he declared that his long course of misconduct was due to the "inheritance of his constitution from a drunken father"; and another habitual offender said that as society manufactured criminals it ought not to blame him, but to answer for him. Anything better calculated to impair and destroy the conscience and sense of rectitude, anything better designed to abolish the sense of responsibility and confirm criminals in their evil ways could scarcely be imagined than teaching of this character. It would be horrible if it were true; it would be disastrous if it rested on any solid ground of fact; it would be pernicious if it were a plausible inference from the facts; but what are we to say of it when we find that it is mere crazy speculation, without any solid ground of fact to support it?

### The Doctrinaire Fanatic.

How logical, how capable of consistent reasoning, these doctrinaires are may be gathered from another instance drawn from the wealth of material in Sir Bryan Donkin's article. A popular writer of fiction argues in a book about prisons that "imprisonment as a punishment has failed." This is when he is writing against imprisonment, and wants to make his point. But when he is writing against flogging he forgets that imprisonment has failed, and says that garrotting was stopped, not by flogging but "by the ordinary law with its ordinary punishments," that is to say, by imprisonment! The fact of the matter is that the reformer wishes to pose as a superior person who knows best. He has no responsibility whatever, and can advocate or decry anything he likes without being brought to book for doing so. In order to pose and to show his superiority he singles out for attack any institution that is long and well established, his reasoning, if the muddy operations of his mind can be called reasoning, being, it seems, that as many well and long established institutions have been found to become faulty when the conditions for which they were created have altered, therefore any and every long and well established institution must be faulty, and ought to be abolished, whether the conditions have altered or not. Whether this is what passes in the mind of the crank as reasoning, or whether he merely wishes to pose as superior without any basis of reasoning at all, does not much matter. The result is the same.

### A Catchword of To-day.

Another word that the amateur criminologist uses to conjure with is "heredity." He does not know what it means, and could not for the life of him explain the difference between heredity and inheritance. But it is highly "scientific," and that suffices. Not many years ago the Legislature of the State of Indiana passed an Act "to prevent the procreation of confirmed criminals, imbeciles, idiots, and rapists." The preamble of this sapient Act began: "Whereas heredity plays an important part in the transmission of crime . . . " as who should say "whereas heredity plays an important part in the transmission of cheese-making," and should enact that women who make good cheese should be compelled to mate with men who can make good bread, so as to procreate children who can make good butter. Fortunately for the population of America, the Acts of State Legislatures are often passed with the tongues of the legislators in their cheeks. The Acts are not put in force, and are never intended to be put in force. They are passed to stop the mouths of cranks and faddists when their outcries become too clamorous to be borne; and when the cranks, who implicitly believe that an Act of Parliament is a cure for every social ill, get their Act they are satisfied, and everything goes on as before, except that the cranks soon begin to clamour for something else.

The fact is manifest enough, and Sir Bryan Donkin is justified in laying strong insistence on it, that law-breaking or "criminality" is no unity; that there are no special qualities, physical or mental, common to all criminals; and that the only important link between the study of crime and that of biological heredity is that a considerably larger minority of persons with clearly appreciable mental defect, apparently of a congenital nature, is found among convicted criminals than in the population at large.

The study of criminals has convinced Sir Bryan Donkin, and no living man is better qualified by intellect, training, and experience to judge, that *all men are potential law-breakers*, and that without the *traditional* (not *biological*) heritage of moral or social experience, human society would be dissolved. The common sense, as shown by the universal practice, of all mankind in all ages has taken, and does take, the same view.

